# Bipedal locomotion, spinal pain and psychiatric disorders. Is this our future? Locomoção bípede, dores na coluna e distúrbios psiquiátricos. É este o nosso futuro?

**DOI:** 10.1590/1516-3180.2016.001220162106

**Published:** 2016-09-26

**Authors:** Isabela Judith Martins Benseñor

**Affiliations:** I MD, PhD. Associate Professor, Department of Internal Medicine, School of Medicine, Universidade de São Paulo (USP), São Paulo, SP, Brazil.

Bipedal locomotion is probably the oldest and one of the most important characteristics of humans. The process of evolution that led to bipedalism is poorly understood, with several knowledge gaps. Is it possible that spinal pain is a consequence of this adaptive process that began a long time ago? Why would this be so important now?

The Global Burden of Disease study 2013 (GBD 2013) published a list of the more common causes of disability-adjusted life-years (DALYs) at three points of time: 1990, 2005 and 2013. DALYs may be understood, in a simple way, as the lost years of "healthy" life.^1^ They are calculated as the sum of the years of life lost (YLL) due to premature mortality in the population and the years lost due to disability (YLD) for people living with an adverse health condition or its consequences. Low-back pain was the seventh biggest cause of DALYs in the world in 1990, the fifth in 2005 and the fourth in 2013. Only three diseases cause more DALYs than low-back pain, according to GBD 2013: ischemic heart disease, stroke and lower respiratory infections.^1^ The situation in Brazil is that low-back pain is the first of YLD and the second biggest cause of DALYs, just behind coronary heart disease (CHD), and followed by violence, stroke, road accidents, diabetes, depression, anxiety, chronic obstructive pulmonary disease and sensory losses.^1^

In this issue of São Paulo Medical Journal, an article is published^2^ that estimates the prevalence of chronic spinal pain in individuals aged 15 years or over, in a sample from a region of the city covered by the Family Health Program. The study also compared quality of life among individuals with and without chronic spinal pain. Chronic spinal pain encompasses neck pain, upper back pain and low-back pain.

What is the importance of this article? It is very simple: if low-back pain is the second biggest cause of DALYs in Brazil, it is time to know more about this symptom and factors associated with it. In the sample, the most important associated factors were sex (women), age, education (low educational attainment), occupational history (physical work) and anxiety symptoms. Psychiatric disorders are a very important cause of chronification of any pain process. The associated factors reported in the analysis published in this issue of the journal are similar to those in previously published studies that showed that psychiatric disorders were common factors associated with low-back pain.^3^

GBD 2013 showed that depressive disorders were the 15^th^ biggest cause of DALYs in 1990, the 14^th^ in 2005 and the 11^th^ in 2013.^1^ In Brazil, depression and anxiety disorders are respectively the seventh and eighth biggest cause of DALYs in our population, according to GBD 2013. Low-back pain, depression and anxiety disorders do not occupy stable positions in the list of the most frequent causes of DALYs. At each new measurement, they have been rising through the rankings. Therefore, we need to be prepared for further ascension over the coming years.

Few studies in Brazil have addressed spinal pain. In a previous systematic review on the prevalence of back pain in Brazil conducted in 2015, it was concluded that there were no representative studies with the capacity to depict the generalizable prevalence of low-back pain in Brazil. The small number of studies available that were included in this review showed a high risk of bias.^4^ Another study published in 2013 calculated the number of years with which individuals lived with low-back pain, using data from the Brazilian Household Sample Survey (PNAD). The results showed that men born in Brazil in 2008 will have a life expectancy of 69.2 years, of which 15% will be spent with chronic spinal diseases; while women born in the same year will have a life expectancy of 76.7 years and can expect to live a fifth of their lives with chronic spinal diseases.^5^

The study published in this issue of the journal not only fills this gap but also brings insights towards better understanding of the burden of spinal pain in Brazil.^2^ Has the price of bipedalism now become inflated? Or are we now more worried about quality of life as a new step towards health? Independent of what the right answer is, this is now the moment to raise a red flag signaling the need for public health policies directed towards adaptation of healthcare services to the increasing frequency of spinal pain and especially low-back pain over the coming years.
